# Puerperal Convulsions

**Published:** 1887-10

**Authors:** Aurel Kraizell

**Affiliations:** Der Allg. Hom. Zeitung


### PUERPERAL CONVULSIONS.

(Aurel Kraizell, M. D., *Der Allg. Hom. Zeitung.*)

Translated by A. McNeil, M. D., San Francisco.

I was called on the forenoon of 21st of December of last year to see in great haste a woman who had been delivered the night before. She was forty years old and had been delivered at midnight. They told me the birth had been accomplished without any bad symptoms. The afterbirth had also passed and no great hemorrhage had occurred. In accordance with a bad custom which prevails in this neighborhood, she had taken soon after delivery some whisky. About one A. M. she became very restless, and soon after was attacked by a violent, general convulsion, with the cry, "Help me!" After this had continued several minutes she fell into an unconscious condition with much groaning and moaning, out of which she awoke in twenty-five or thirty minutes to be again attacked. The paroxysms had been constantly returning since ten o'clock, and appeared to be increasing in violence, and for several hours she had given no indication of consciousness. As I entered her room I *witnessed the sixteenth eclamptic attack of the highest grade of violence,* which

I was told by those present had already continued almost a half hour. All the muscles of the body were affected by the most violent clonic spasms, so that three men must hold her to prevent her from throwing herself out of the bed. Her face was unrecognizably distorted and of a bluish red color, the eyeballs extravasated with blood, pupils dilated, and out of her mouth poured a bloody, frothy saliva. Respiration was very difficult, in jerks, with rattling on the chest, the heart beat violently and irregularly. The abdomen was very much distended, and through its walls the spasmodic movements of the intestines were perceptible to the hand. The temperature of the skin of the entire body, but particularly of the head, was very much increased, the pulse imperceptible. The entirely unconscious patient kept up a dismal groaning.

I ordered her mouth to be cleansed as well as possible from blood and mucus with a linen rag, and placed two pellets of Belladona$^{30c}$ on her tongue. In a few minutes the convulsions had abated and she had fallen into an unconscious condition, while she broke out into a profuse clammy sweat. I now dissolved some pellets of the same medicine in a glass of water, and with great difficulty administered a teaspoonful of this solution, as swallowing was nearly impossible. Of this she received a spoonful at first every half hour and afterward at longer intervals, with the result that she had no more eclamptic attacks, and the consciousness gradually returned that evening.

On the next day, to my great joy, I found the supposed dying woman fully conscious, and complaining only of violent pains in her head and groins. The skin of her entire body was in a uniform perspiration, the pulse regular and undulating, the abdomen soft and not sensitive on pressure, and the lochia had returned. The medicine was continued every two hours. On the following day the pains in the head and groins had abated, and under the administration of the Belladonna solution at longer intervals she passed safely through the dangers of the lying-in room.

